# Academic Orthopaedic Surgeons Prefer Peer-Reviewed Guidelines Over Artificial Intelligence and Social Media for Medical Information

**DOI:** 10.7759/cureus.113545

**Published:** 2026-07-28

**Authors:** Divya Bhatia, Melissa Romoff, Emily Tse, Asha Timm, Michael S Kim, Emily Mills, Hao-Hua Wu, Sohaib Hashmi, Don Park, Yu-Po Lee

**Affiliations:** 1 Research, Palos Verdes High School, Palos Verdes Estates, USA; 2 Department of Orthopaedic Surgery, University of California, Irvine, School of Medicine, Orange, USA; 3 Fielding School of Public Health, University of California, Los Angeles, Los Angeles, USA

**Keywords:** artificial intelligence in medicine, effects of social media, large language model, orthopaedic surgery, science communication

## Abstract

Background and objective

Patients increasingly seek recommendations for improved health and wellness, as well as medical advice, through social media and large language models (LLMs), yet the quality of these sources varies considerably. Effective clinical communication requires an understanding of how patients engage with online medical content, as these sources may shape expectations even before a clinical encounter. However, to date, no study has assessed how orthopaedic surgeons themselves use these same resources. Hence, this study aimed to evaluate orthopaedic surgeons' self-reported use of LLMs, social media, and peer-reviewed clinical resources for patient care and personal medical care, along with the perceived understandability and helpfulness of each source.

Methods

In this cross-sectional study, an electronic survey was administered to a convenience sample of all orthopaedic surgeons at a single academic institution. Respondents indicated whether they had ever used LLMs (e.g., ChatGPT), social media platforms (e.g., TikTok, Instagram), or peer-reviewed clinical resources (e.g., guidelines from the North American Spine Society (NASS)) for patient care or their own medical care. Those who reported use were asked whether each source was easy to understand and helpful. Proportions are reported with 95% Wilson confidence intervals (CIs). McNemar's exact test was used to compare paired utilization rates across sources and contexts, while Fisher's exact test was employed to compare comprehensibility between distinct user subgroups. All analyses were exploratory given the pilot design.

Results

Sixteen orthopaedic surgeons completed the survey. Peer-reviewed clinical guidelines were used by 15 surgeons (93.8%; 95% CI: 71.7%-98.9%) for patient care and 14 (87.5%; 95% CI: 64.0%-96.5%) for personal care, significantly more frequently than LLMs or social media for both contexts (all p ≤ 0.016). LLMs were used by five surgeons (31.2%; 95% CI: 14.2%-55.6%) for patient care and seven (43.8%; 95% CI: 23.1%-66.8%) for personal care. LLM use for personal care was significantly more frequent than social media use (p = 0.031). Social media use was rare, reported by two surgeons (12.5%) for patient care and one (6.2%) for personal care. Every surgeon who used an LLM in either context rated it as helpful, and the majority found it easy to understand. Comprehensibility ratings did not differ significantly between LLM users and guideline users for either patient care (80.0% vs. 66.7%; p = 1.000) or personal care (85.7% vs. 64.3%; p = 0.613). Concordance analysis showed that 14 of 16 surgeons used guidelines in both contexts, whereas eight had never used an LLM and 14 had never used social media in either setting.

Conclusions

Academic orthopaedic surgeons demonstrate a strong and statistically significant preference for peer-reviewed clinical guidelines over LLMs and social media for both personal and patient medical care. LLMs occupy an intermediate position in surgeons' information-seeking preferences and were rated as helpful by all users, suggesting cautious but growing adoption. Social media use for medical purposes was rare among surgeons, contrasting sharply with published estimates exceeding 70% among the general public. This disconnect, with patients seeking orthopaedic information on platforms where expert content is scarce, represents a clinically meaningful gap.

## Introduction

Patients increasingly seek and encounter medical information outside the clinical setting through social media platforms and large language models (LLMs) [1]. While these tools may improve access and provide simple and comprehensible explanations of medical topics, they also expose patients to content that may not be vetted or reviewed by medical professionals [2,3]. Social media content may be anecdotal, unsourced, or misleading, whereas LLM-generated responses may be limited by insufficient clinical context, variable specificity, and the need for physician interpretation before being applied to patient care [4]. In modern practice, effective clinical communication requires an understanding of how patients obtain and engage with online medical information, as these sources shape patient expectations even prior to seeking medical attention [5].

These concerns are particularly relevant in orthopaedic surgery, where patients frequently seek information regarding common musculoskeletal problems, orthopaedic procedures, and postoperative expectations [6,7]. Although orthopaedic content is widely available on social media, prior analysis of popular orthopaedic surgery videos on TikTok found that most videos were of low quality according to DISCERN criteria, largely due to limited reliability, sourcing, and discussion of both the potential benefits and risks of treatment options [8]. Similarly, studies of LLM use in spine and orthopaedic patient education suggest that artificial intelligence (AI)-generated responses may serve as useful supplemental educational tools, but their limitations are most apparent when questions require individualized counseling, postoperative expectation-setting, or adaptation to a patient’s health literacy and clinical context [9].

While prior studies have separately evaluated the quality of online health content or patient information-seeking behavior, to date, no study has directly compared how academic orthopaedic surgeons themselves utilize AI LLMs, social media, and traditional peer-reviewed resources across both clinical practice and personal care contexts. Therefore, the primary objective of this study was to evaluate and compare the self-reported utilization rates of these distinct information sources among academic orthopaedic surgeons. Secondary objectives included assessing and comparing the perceived understandability and helpfulness of each source within these dual contexts, thereby establishing foundational baseline data regarding professional digital adoption and the broader clinical communication gap.

## Materials and methods

Study design and ethical considerations

This was a cross-sectional survey study designed to evaluate self-reported use of AI LLMs, social media platforms, and peer-reviewed clinical resources for medical information among orthopaedic surgeons. The study was reviewed by the University of California, Irvine Institutional Review Board and was determined to qualify for exempt status (STUDY00001622). No patient-level data were collected.

Participants and survey administration

All orthopaedic surgeons at a single academic institution were surveyed. Participation was entirely voluntary and anonymous; survey response and completion constituted implied consent. Sixteen orthopaedic surgeons, all of whom hold Doctor of Medicine (MD) degrees, completed the survey and were included in the final analysis.

Survey instrument

The survey assessed whether respondents had ever used three categories of medical information sources: AI LLMs, social media platforms, and peer-reviewed clinical resources. These sources were evaluated in two contexts: the respondent’s own medical care and the care of a patient.

The survey evaluated three broad categories of digital and traditional resources: AI LLMs (with ChatGPT/GPT-4o provided as a primary example), social media platforms (explicitly referencing TikTok and Instagram), and peer-reviewed clinical guidelines (including formal literature and society-based recommendations such as those from the North American Spine Society (NASS)). While specific flagship tools were provided as representative anchors to standardize participant interpretation, responses reflected aggregate utilization of each technological class rather than an exclusive evaluation of any single platform.

For each information source and care context, respondents were asked whether they had ever used that source. Respondents who answered affirmatively were then asked whether they found the source easy to understand and whether they found it helpful. The complete survey instrument is provided in the Appendices.

Outcomes

The primary outcome was the proportion of surgeons reporting prior use of each information source for personal medical care and for patient care. Secondary outcomes included the proportion of users who rated each source as easy to understand and helpful.

Statistical analysis

Categorical variables were summarized as counts and percentages, and 95% confidence intervals (CIs) were calculated for all proportions using the Wilson method. The Wilson method was selected over the standard Wald interval because it performs better in small samples and when proportions are near zero or one, where Wald intervals can be inaccurate or fall outside the 0%-100% range. Because the same 16 surgeons responded to questions about all three information sources, their responses across sources are correlated by design. For this reason, pairwise comparisons of utilization rates between sources were performed using McNemar’s exact test rather than Fisher’s exact test, which assumes two independent groups. The same approach was applied when comparing utilization between personal and patient care contexts within each source. Fisher’s exact test was used to compare understandability ratings between LLM users and guideline users, as these represent independent subgroups.

Given the pilot nature of the study and the small sample size, all analyses were considered exploratory and hypothesis-generating. A two-sided significance threshold of p < 0.05 was applied. All analyses were performed using R (version 4.3.1; R Foundation for Statistical Computing, Vienna, Austria). Wilson CIs were calculated using the PropCIs package. McNemar’s exact test was performed using the exact 2x2 package. Fisher’s exact test was performed using base R functionality.

## Results

Participant characteristics

Sixteen academic orthopaedic surgeons completed the survey. All 16 respondents held an MD degree and were actively engaged in orthopaedic surgery practice.

Utilization of information sources for patient care

Five surgeons (31.2%; 95% CI: 14.2%-55.6%) reported having used an LLM in the care of a patient (Table 1). Two surgeons (12.5%; 95% CI: 3.5%-36.0%) had referenced social media in patient care. This difference from LLM use was not statistically significant (discordant pairs: 4 vs. 1; p = 0.375, McNemar’s exact test) (Table 2). Peer-reviewed guidelines were used for patient care by 15 surgeons (93.8%; 95% CI: 71.7%-98.9%), significantly more frequently than LLM use (discordant pairs: 0 vs. 10; p = 0.002) and social media use (discordant pairs: 0 vs. 15; p < 0.001) (Table 2).

**Table 1:** Utilization, comprehensibility, and helpfulness by information source among 16 academic spine surgeons AI: artificial intelligence; LLM: large language model; CI: confidence interval

Information source	Context	Used, n (%)	95% CI	Easy to understand, n/used (%)	Helpful, n/used (%)
AI LLM	Personal care	7 (43.8%)	23.1%-66.8%	6/7 (85.7%)	7/7 (100%)
	Patient care	5 (31.2%)	14.2%-55.6%	4/5 (80.0%)	5/5 (100%)
Social media	Personal care	1 (6.2%)	1.1%-28.3%	1/1 (100%)	0/1 (0%)
	Patient care	2 (12.5%)	3.5%-26.0%	2/2 (100%)	1/2 (50.0%)
Peer-reviewed guidelines	Personal care	14 (87.5%)	64.0%-96.5%	9/14 (64.3%)	14/14 (100%)
	Patient care	15 (93.8%)	71.7%-98.9%	10/15 (66.7%)	15/15 (100%)

**Table 2:** Pairwise McNemar’s exact test results for utilization comparisons (N = 16 paired observations) McNemar’s exact test was used throughout, given the small sample size. For personal and patient care comparisons, discordant pairs represent surgeons who used one source but not the other. For within-source comparisons, discordant pairs represent surgeons whose personal and patient care differed AI: artificial intelligence; LLM: large language model

Comparison	Context	Test	P-value
Personal care comparisons			
AI LLM vs. social media	Personal care	McNemar	0.031
AI LLM vs. peer-reviewed guidelines	Personal care	McNemar	0.016
Social media vs. peer-reviewed guidelines	Personal care	McNemar	< 0.001
Patient care comparisons			
AI LLM vs. social media	Patient care	McNemar	0.375
AI LLM vs. peer-reviewed guidelines	Patient care	McNemar	0.002
Social media vs. peer-reviewed guidelines	Patient care	McNemar	< 0.001
Own vs. patient care (same source)			
AI LLM: own care vs. patient care	Within source	McNemar	0.625
Social media: own care vs. patient care	Within source	McNemar	1
Guidelines: own vs. patient care	Within source	McNemar	1

Among the five surgeons who used an LLM for patient care, four (80.0%; 95% CI: 37.6%-96.4%) found it easy to understand, and all five (100%; 95% CI: 56.6%-100%) found it helpful (Table 1). Both surgeons who referenced social media in patient care found it easy to understand. One found it helpful, and one did not. Among the 15 surgeons who used guidelines for patient care, 10 (66.7%; 95% CI: 41.7%-84.8%) found them easy to understand, and all 15 (100%; 95% CI: 79.6%-100%) found them helpful (Table 1). The comprehensibility rating did not differ significantly between LLM users and guideline users for patient care (80.0% vs. 66.7%; p = 1.000, Fisher’s exact test).

Utilization of information sources for personal medical care

Seven of 16 surgeons (43.8%; 95% Wilson CI: 23.1%-66.8%) reported using an LLM such as ChatGPT for their own medical care. Only one surgeon (6.2%; 95% CI: 1.1%-23.8%) reported using social media for personal medical care (Table 1). According to McNemar’s exact test, LLM use was significantly more frequent than social media use for personal care (discordant pairs: 6 vs. 0; p=0.031). Peer-reviewed clinical guidelines were used for personal medical care by 14 surgeons (87.5%; 95% CI: 64.0%-96.5%), significantly more frequently than both LLM use (discordant pairs: 0 vs. 7; p=0.016) and social media use (discordant pairs: 0 vs. 13; p<0.001) (Table 2).

Among the seven surgeons who used an LLM for personal care, six (85.7%; 95% CI: 48.7%-97.4%) found it easy to understand, and all seven (100%; 95% CI: 64.6%-100%) found it helpful (Table 1). The single surgeon who used social media for personal medical care found it easy to understand, but not helpful. Among the 14 surgeons who used peer-reviewed guidelines for personal care, nine (64.3%; 95% CI: 38.8%-83.7%) found them easy to understand, and all 14 (100%; 95% CI: 78.5%-100%) found them helpful (Table 1). Comprehensibility ratings did not differ significantly between LLM users and guideline users for personal care (85.7% vs. 64.3%; p=0.613; Fisher’s exact test).

Within-source consistency: personal vs. patient care use

For each information source, McNemar’s exact test was used to assess whether utilization differed between personal and patient care contexts among the same surgeons. No significant differences were observed for LLMs (discordant pairs: 3 vs. 1; p=0.625), social media (discordant pairs: 0 vs. 1; p=1.000), or peer-reviewed guidelines (discordant pairs: 0 vs. 1; p=1.000) (Table 2). Concordance patterns are shown in Table 3. Peer-reviewed guidelines showed the highest concordance across contexts, with 14 of 16 surgeons (87.5%) using them for both personal and patient care. By contrast, eight surgeons (50.0%) reported never using an LLM in either context, and 14 (87.5%) reported never using social media in either context.

**Table 3:** Concordance of information source use between personal and patient care contexts (N = 16) Values represent the number of surgeons (%) in each concordance category AI: artificial intelligence; LLM: large language model

	Used for both	Personal care only	Patient care only	Used for neither
AI LLM	4 (25.0%)	3 (18.8%)	1 (6.2%)	8 (50.0%)
Social media	1 (6.2%)	0 (0%)	1 (6.2%)	14 (87.5%)
Peer-reviewed guidelines	14 (87.5%)	0 (0%)	1 (6.2%)	1 (6.2%)

## Discussion

Academic orthopaedic surgeons in this cohort demonstrated a clear and statistically significant preference for medical information sources. Peer-reviewed clinical guidelines were used by 87.5% of surgeons for personal medical care and 93.8% for patient care, significantly more frequently than either LLMs or social media in both contexts. LLMs occupied a middle position, with 43.8% of surgeons using them for personal care and 31.2% for patient care, and were rated as helpful by every surgeon who used them. Social media use was rare, with only one surgeon using it for personal care and two for patient care, and it was significantly less frequent than guideline use in both contexts.

These findings contrast sharply with published data on how the general public seeks health information. Up to 72% of American adults use social media for health-related questions [10], and studies of patients presenting to spine clinics report online health information-seeking rates as high as 96% [8]. In the present cohort, only one surgeon (6.2%) used social media for personal medical care, a gap of more than 60 percentage points relative to general population estimates. This disparity is consistent with published data showing that fewer than 15% of spine surgeons maintain an active patient-facing social media presence [11,12], and that surgeon-generated content represents a small minority of popular orthopaedic-related posts on platforms such as TikTok [13-15]. As a result, the orthopaedic-related content most readily available to patients on these platforms is generated primarily by non-surgeons, including chiropractors, influencers, and laypersons, whose content has been shown to contain substantial rates of misinformation [8,13-15].

The finding that 43.8% of academic orthopaedic surgeons reported using an LLM for personal medical care is among the more interesting observations of this study. Prior work has shown that ChatGPT-generated spine advice is perceived as more accurate than TikTok content, though less reliable than NASS guidelines [16], and that LLMs such as ChatGPT can meaningfully improve the readability of orthopaedic patient education materials to better meet American Medical Association (AMA)-recommended reading levels [17]. The present data extend these findings. Nearly half of the surgeons surveyed had already used an LLM for their own medical care, and every surgeon who did so found it helpful. This pattern suggests that LLM adoption among orthopaedic surgeons may be more prevalent than previously reported, though larger multi-institutional studies are needed to confirm this. Comprehensibility did not differ significantly between LLM users and guideline users in either context (p = 0.613 for personal care; p = 1.000 for patient care), suggesting that surgeons who engage with AI tools find them comparably accessible to formal clinical guidelines.

The concordance data add further context. Peer-reviewed guidelines showed the strongest behavioral consistency: 14 of 16 surgeons used them in both personal and patient care contexts, and only one used them in neither. LLMs showed a more mixed pattern, as four surgeons used them in both contexts, three used them for personal care only, one used them for patient care only, and eight used them in neither, consistent with a tool that some surgeons have integrated into their practice while others have not yet adopted. Social media was used in both contexts by only one surgeon and in neither context by 14, reflecting near-universal non-adoption for medical care purposes.

This pilot study has significant limitations, particularly because of the limited convenience sample of 16 surgeons from a single academic institution. Consequently, statistical power is constrained, confidence intervals are wide, and the findings cannot be generalized to orthopaedic surgeons in community practice or in other geographic regions. Furthermore, the reliance on self-reported data without granular task-specific workflows introduces potential ambiguity regarding the exact clinical applications of large language models. These limitations underscore that our findings are strictly hypothesis-generating, establishing a methodological framework and preliminary estimates to guide future large-scale, multi-institutional investigations.

Furthermore, our survey relied entirely on self-reported responses and lacked granularity regarding specific task workflows. For example, asking whether an LLM was used for patient care does not distinguish whether it was used for drafting clinical notes, translating discharge instructions into accessible reading levels, summarizing complex literature, or assisting with diagnostic decision-making, distinctions that carry vastly different clinical implications. Future multi-institutional studies should incorporate branched survey logic or qualitative task inventories to determine whether surgeon adoption is primarily driven by administrative efficiency, educational drafting, or clinical decision support.

## Conclusions

Academic orthopaedic surgeons demonstrate a strong and statistically significant preference for peer-reviewed clinical guidelines over LLMs and social media for both personal and patient medical care. LLMs occupy an intermediate position in surgeons’ information-seeking preference and were rated as helpful by all users, suggesting growing but selective adoption that may accelerate as institutional guidance matures. Social media use for medical care was rare among surgeons, standing in stark contrast to published rates of social media health information-seeking among the general public that exceed 70%. This gap represents a clinically meaningful informational dissonance: patients are seeking orthopaedic health information on platforms where credentialed expert content may be saturated by that of influencers and laypersons. These findings are limited by a small single-institution sample and should be interpreted as hypothesis-generating. Future large-scale, multi-institutional studies are needed to confirm these patterns, characterize how AI tools are being used in clinical contexts, and evaluate strategies for increasing expert surgeon engagement on patient-facing platforms to improve the quality and accuracy of publicly available orthopaedic health information.

## Appendices

Appendix: survey

Please answer the questions below. Thank you for your participation.

1. Have you ever used an artificial intelligence engine (for example, ChatGPT, etc.) for your own medical care?

a. If so, did you find this source to be easy to understand?

b. If so, did you find this source to be helpful?

2. Have you ever used an artificial intelligence engine (for example, ChatGPT, etc.) for the care of a patient?

a. If so, did you find this source to be easy to understand?

b. If so, did you find this source to be helpful?

3. Have you ever used social media (for example, TikTok, Instagram, etc.) for your own medical care?

a. If so, did you find this source to be easy to understand?

b. If so, did you find this source to be helpful?

4. Have you ever used social media (for example, TikTok, Instagram, etc.) for the care of a patient?

a. If so, did you find this source to be easy to understand?

b. If so, did you find this source to be helpful?

5. Have you ever used peer-reviewed research for your own medical care?

a. If so, did you find this source to be easy to understand?

b. If so, did you find this source to be helpful?

6. Have you ever used peer-reviewed research for the care of a patient?

a. If so, did you find this source to be easy to understand?

b. If so, did you find this source to be helpful?
